# Investigation on the relationship between hemoglobin concentration and stroke risk: a bidirectional Mendelian randomization study

**DOI:** 10.3389/fneur.2024.1327873

**Published:** 2024-04-25

**Authors:** Wenbao Wu, Daofeng Fan, Binfu Que, Yangui Chen, Rui Qiu

**Affiliations:** ^1^Department of Acupuncture and Moxibustion, Longyan First Hospital Affiliated to Fujian Medical University, Longyan, China; ^2^Department of Neurology, Longyan First Hospital Affiliated to Fujian Medical University, Longyan, China

**Keywords:** two-sample Mendelian randomization, hemoglobin concentration, ischemic stroke, stroke, Cardioembolic stroke

## Abstract

**Background:**

The relationship between hemoglobin concentration and stroke has garnered significant interest in the research community. However, findings from published observational epidemiological studies on this relationship have been inconclusive. By using publicly available genome-wide association study (GWAS) aggregated statistics, a two-sample Mendelian randomization analysis is conducted to explore the causal relationship between hemoglobin concentration and stroke.

**Methods:**

Summary statistics data from UK Biobank for hemoglobin concentration and from the FinnGen R9 and MEGASTROKE consortium for stroke are used. A series of quality control steps are taken to select eligible instrumental SNPs closely related to exposure. In order to make the conclusion more robust and reliable, several robust analysis methods are employed including inverse variance weighted, weighted median, MR-Egger regression, which are based on different assumptions of two-sample MR Analysis. Meanwhile, sensitivity analyses such as pleiotropy test and MR-Egg regression, are performed to mitigate horizontal pleiotropy and heterogeneity.

**Results:**

The two-sample Mendelian randomized study indicates a negative association between hemoglobin concentration and stroke, suggesting that hemoglobin concentration acts as a protective factor against stroke. From the FinnGen database, there is a negative association between hemoglobin concentration and stroke, with an odds ratio (OR) of 0.82 and a 95% confidence interval (CI) of 0.73–0.92, *p* = 0.0006. Similarly, the MEGASTROKE database findings reinforce this observation. The negative association between hemoglobin concentration and stroke (OR: 0.91, 95%CI: 0.83–1.00, *p* = 0.040), ischemic stroke (OR: 0.87, 95%CI: 0.79–0.96, *p* = 0.004), and cardiogenic stroke (OR: 0.82, 95% CI: 0.69–0.99, *p* = 0.039) further suggests that higher hemoglobin levels might confer a protective effect against these conditions.

**Conclusion:**

Hemoglobin concentration serves as a protective factor against stroke, and managing abnormal hemoglobin levels can effectively reduce the incidence of stroke.

## 1 Introduction

Stroke, a leading causes of death and long-term disability, continues to pose a significant global health burden. Although advancements in secondary stroke prevention and diagnostic and treatment protocols have contributed to minimizing acute ischemic stroke occurrences, the incidence of stroke remains high (1). Effective interventions targeting stroke risk factors have proven successful in reducing stroke incidence (2). Traditionally, various cardiovascular risk factors, such as hypertension, diabetes, and hyperlipidemia have been closely associated with the development of stroke (3). However, with the advancement of research as well as technology, new biomarkers have attracted the attention of scientists, including hemoglobin concentration.

The relationship between hemoglobin concentration and stroke is still of interest, but it remains uncertain. Observational studies have demonstrated a complex relationship between hemoglobin concentration and stroke (4). High hemoglobin concentration is seen in conditions like polycythemia vera (5), chronic obstructive pulmonary disease (6) and plateau erythrocytosis (7). Numerous studies have shown that high hemoglobin concentrations increased the blood clotting tendencies and thrombosis risk. Clinical evidence has linked polycythemia vera to thrombosis, including stroke (8), suggesting that high hemoglobin concentration is a risk factor for stroke. Hemoglobin is a key oxygen-carrying molecule in the blood, the relationship between stroke and anemia can be partially explained by a direct link between the central nervous system, blood supply, and oxygen delivery to tissues (9). Consequently, anemia is also a risk factor for ischemic stroke and is associated with a higher mortality rate after hospitalization (10). The relationship between anemia and increased mortality or disability in patients with various types of stroke, including ischemic stroke, cerebral hemorrhage, and subarachnoid hemorrhage, has been investigated (11). However there is inconclusive evidence regarding the correlation, positive or negative, between hemoglobin concentration and stroke.

Randomized controlled Trails (RCTs) are the gold standard for establishing causal relationships in epidemiological studies. However, due to medical ethical restrictions and high costs, conducting certain RCTs can be challenging. In contrast, observational studies are widely used in initial causal exploration due to their relatively simple design and ease of implementation. However, confounding factors and inversion of causality often limit the ability to infer causality. Mendelian randomization (MR) principles address these challenges by utilizing genotypes as instrumental variables to investigate genetic traits and their associations, thereby allowing for the study of genetic interactions and causal inference (12). Therefore, genotypes can be used as instrumental variables to study intermediate phenotypes to infer causal associations with disease states, avoiding the influence of confounding factors and reverse causal associations (13). The rapid development of genome-wide association studies (GWAS) has led to the increasing application of MR analysis, using single nucleotide polymorphisms (SNPs) that are strongly correlated with phenotypes as instrumental variables (12).

In the present study, MR analyses are performed to elucidate whether hemoglobin concentration has a causal effect on stroke. Specifically, correlational MR analyses are conducted to investigate the relationship between hemoglobin concentration and stroke, utilizing hemoglobin concentration as an exposure tool for genetic variation and stroke as the outcome. The aim was to obtain causal estimates and determine whether hemoglobin concentration is negatively or positively associated with stroke.

## 2 Methods

### 2.1 Study design

**Figure 1:**
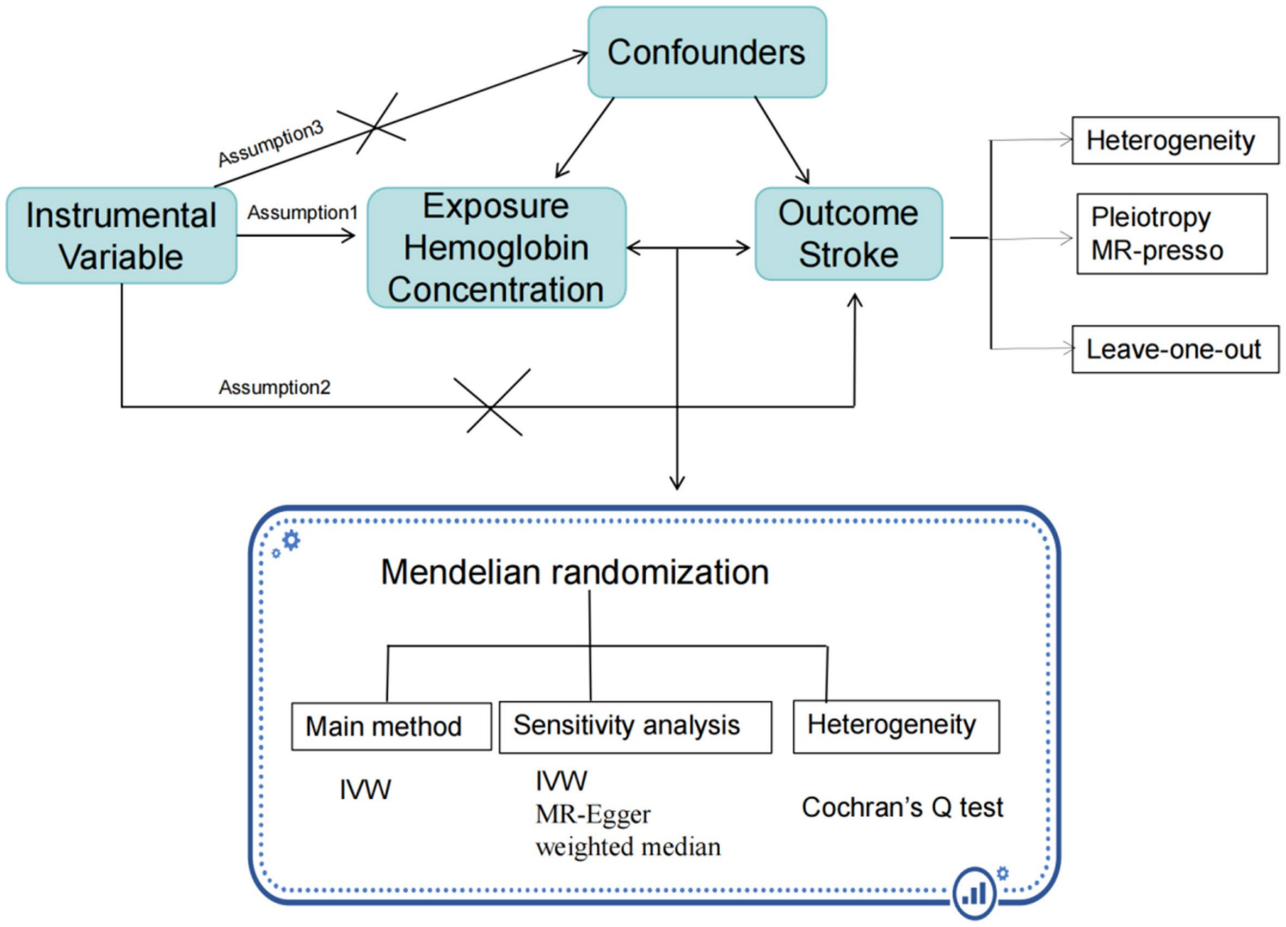
The study flow chart.

A two-sample MR approach is employed using summary statistics data from UK Biobank for hemoglobin concentration, and from the FinnGen R9 and MEGASTROKE consortium for stroke. The use of these datasets was in accordance with appropriate patient consent and ethical approval. The present study was approved by the ethics committee of Longyan First Hospital (Ethics number: 2022022). The specific process is illustrated in the Figure 1.

### 2.2 Outcome data sources

Summary-level data for stroke were obtained from the FinnGen R9 and MEGASTROKE, FinnGen R9 consortium which including a total of 306,377 individuals of European ancestry, consisting of 39,818 stroke cases and 271,817 controls (13). Moreover, in the MEGASTROKE Consortium’s meta-analysis of genome-wide association study (GWAS) data, stroke data were collected, which included stroke subtypes and stroke information in Europeans (40,585 cases, 406,111 controls) (14). The sources and detailed information of this data are presented in Table 1.

**Table 1:** Stroke subtypes and data sources.

Outcome	Sample size (cases/controls)	Ancestry	Significance level	Data sources
Stroke	39818/27181	European	5e−8	https://www.finngen.fi/fir9.finngen.fi
Stroke	40585/406111	European	5e−8	MEGASTROKE Consortium (ebi-a-GCST005838)
Ischemic stroke	34217/406111	European	5e−8	MEGASTROKE Consortium (ebi-a-GCST005843)
Cardioembolic stroke	7193/406111	European	5e−8	MEGASTROKE Consortium (ebi-a-GCST005842)
Large artery stroke	4373/406111	European	5e−8	MEGASTROKE Consortium (ebi-a-GCST005840)
Small vessel stroke	5386/192662	European	5e−8	MEGASTROKE Consortium (ebi-a-GCST005841)

### 2.3 Instrumental variable selection

Hemoglobin concentration as exposure data (containing 13,791,467 SNPs) in a population of 350,474 European ancestry is selected from the UK Biobank dataset (15). Among these SNPs, 71,104 SNPs are identified robustly associated with hemoglobin concentration (*p* < 5×10–8) (16). To ensure the independence of the hemoglobin concentration instrumental variables, we applied clumping with an r2 threshold<0.001 and a clump window of 10,000 kb based on the 1,000 genomes linkage disequilibrium (LD) reference panel of only Europeans (17). After implementing the correlation settings, we identified 287 SNPs that were used as instrumental variables for the exposure. We calculated the F-statistics of these 287 SNPs to assess the strength of genetic variation, and all F-statistics were found to be more than 10. Details of the SNPs associated with the hemoglobin concentration are presented in Supplementary Table S1. 255 and 275 exposure SNPs were obtained from the instrumental variables for stroke outcome data in Supplementary Tables S2, S3. After conducting a harmonize check, we removed some SNPs due to palindromic alleles and compatibility issues with other alleles. After the above operations, we obtained SNPS suitable for MR analysis, which are detailed in Supplementary Tables S4, S5.

### 2.4 Pleiotropy assessment

To identify and exclude possible pleiotropic associations between instrumental variables and other phenotypes, all SNPs are searched using the human genotype–phenotype association database (PhenoScanner V2)^1^ to detect possible pleiotropy (18). Through this analysis, it’s found that that some of the SNPs related to hemoglobin concentration were also associated with various stroke risk factors, including hypertension, diabetes, dyslipidemia, heart disease, smoking, alcohol consumption, overweight or obesity, platelet aggregation, etc. By controlling for confounding factors that may affect stroke outcomes, specific SNPs can be identified to serve as instrumental variables. Detailed information regarding these SNPs can be found in Supplementary Tables S6–S11.

### 2.5 Statistical analysis

To assess the associations between hemoglobin concentration and stroke, multiple statistical methods including inverse variance weighted (IVW), MR-Egger regression, weighted median approaches were used to examine the potential causal association. The IVW method uses a meta-analysis approach combined with Wald estimates for each SNP, to estimate the causal relationship between hemoglobin concentration and stroke (12). In the absence of horizontal pleiotropy, the IVW method provides unbiased results. The weighted median method weights the estimates of causal effects at each locus of genetic variation and enhance the accuracy and robustness of causal inference (19). The MR-Egger regression method corrects for horizontal pleiotropy by estimating the bias weight, which enhances the accuracy and reliability of Mendelian randomization analysis (20). We employed both the MR-Egger method and outlier (MR-PRESSO) method to test for horizontal pleiotropy. Furthermore, a ‘leave one out’ analysis was conducted to examine whether the causal relationship between exposure and outcome was influenced by a single SNP. Odds ratios (ORs) for stroke were calculated per one standard deviation (SD) increase in genetically predicted hemoglobin concentration in all analyses. All SNPs that had a significant effect on hemoglobin concentration at the genome-wide significance levels were used for sensitivity analysis. All statistical analyses were two-sided and performed in R 4.3.0 software (R Foundation for Statistical Computing, Vienna, Austria). MR analyses were performed using the TwosampleMR (version 0.5.7), Mendelian Randomization (version 0.5.7), and MR-PRESSO (version 1.0) packages. *P* < 0.05/5 (with Bonferroni corrections) was statistically significant with *p*-value between 0.05 and 0.01 as suggestively significant. We interpreted the results not solely based on *p*-values but also considered the strengths of the associations and the consistency across sensitivity analyses.

## 3 Results

### 3.1 Exploration of the causal relationship between hemoglobin concentration and stroke

**Figure 2:**
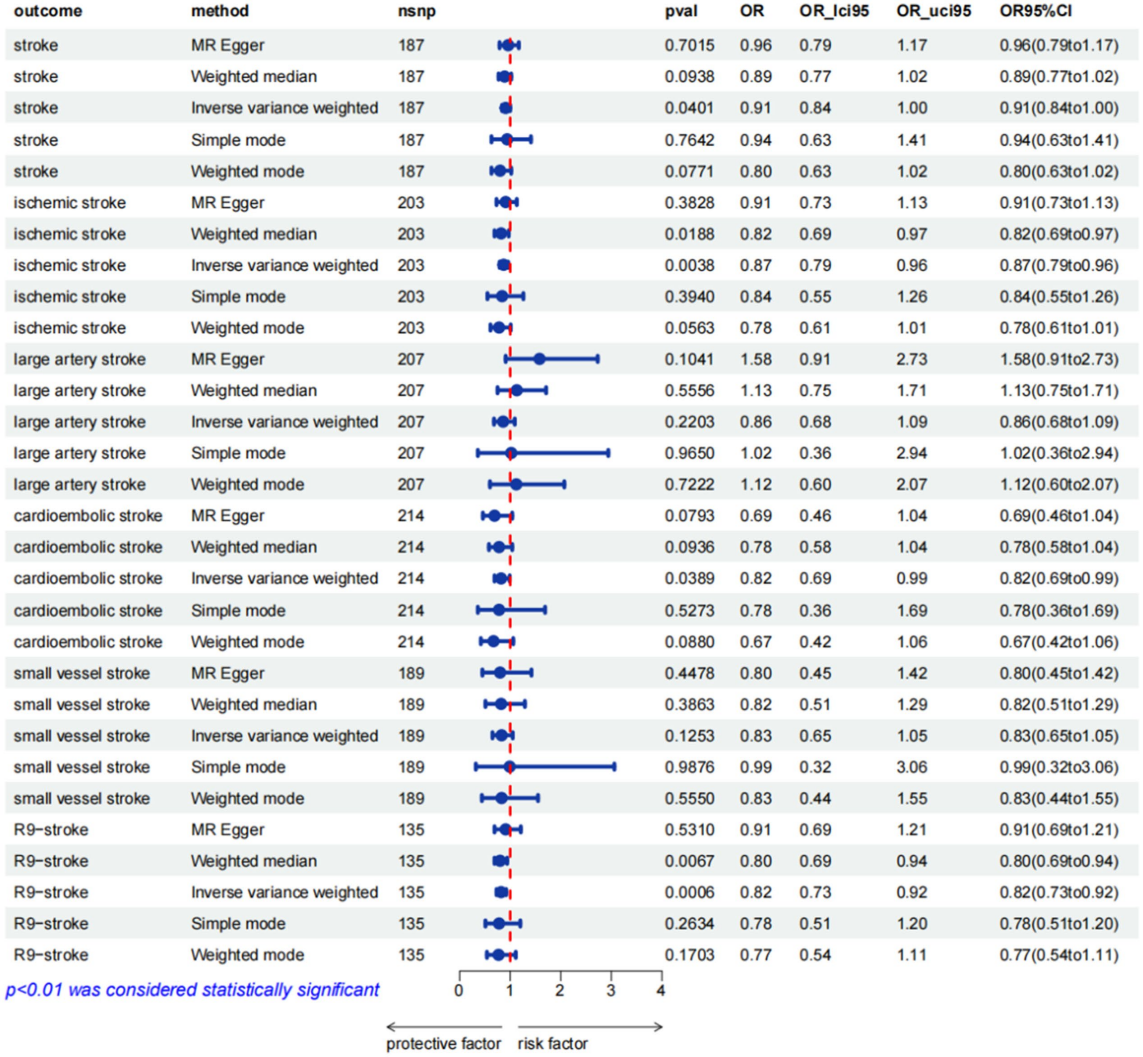
MR estimates from different methods for assessing the causal effect of hemoglobin concentration on stroke (pval was the value pval after MRpresso).

**Figure 3:**
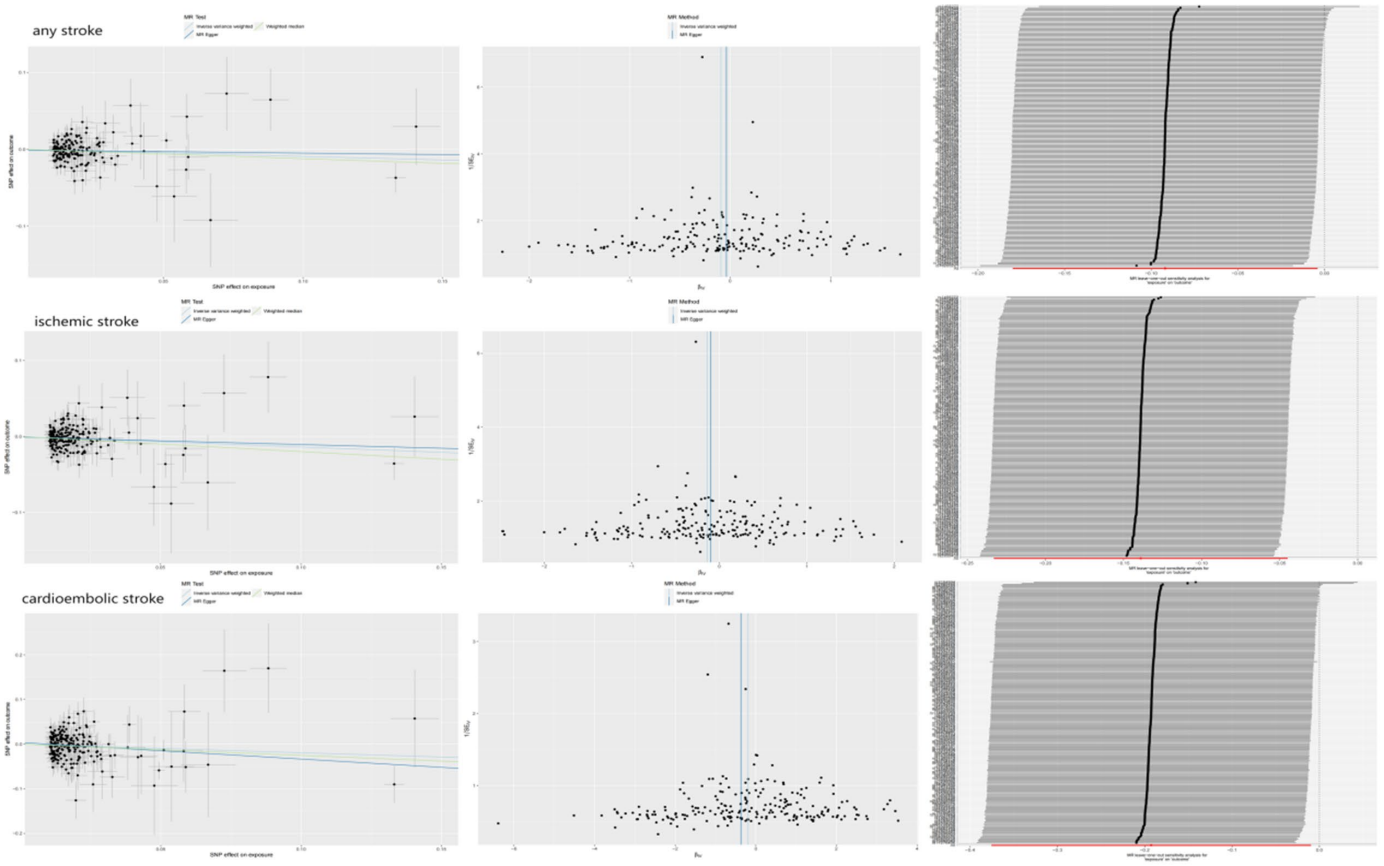
The causal effect of hemoglobin concentration on stroke (MEGASTROKE).

**Figure 4:**
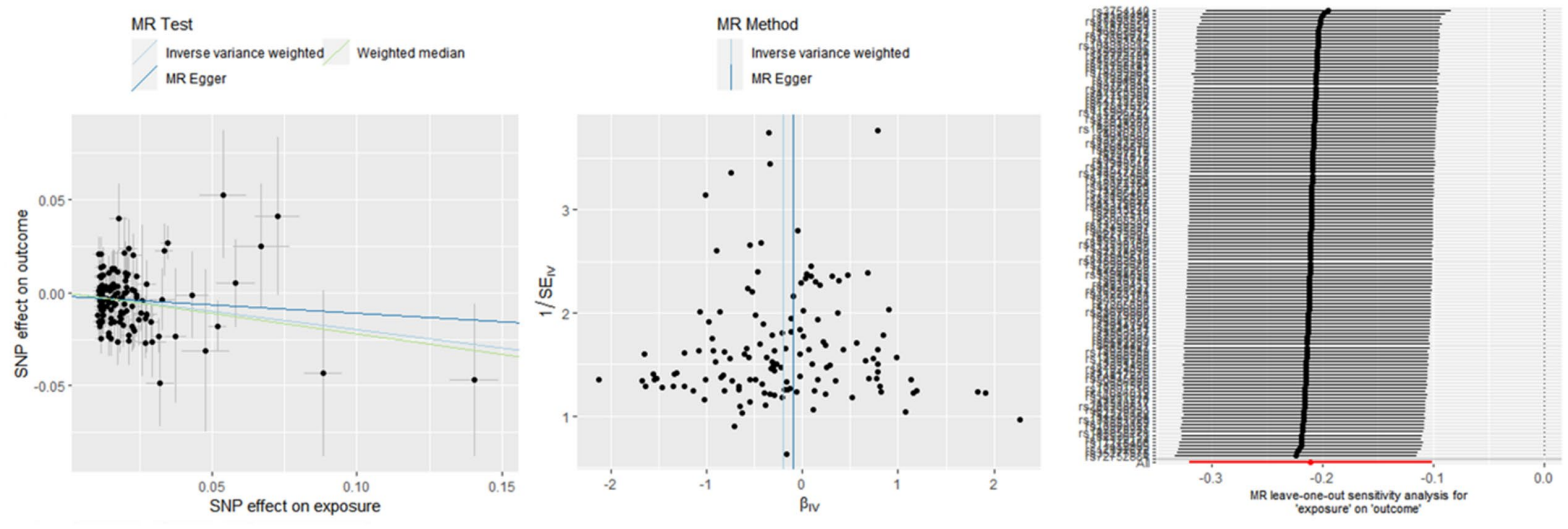
The causal effect of hemoglobin concentration on stroke (FinnGen R9).

The associations between hemoglobin concentration and stroke are shown in Figure 2. This study found a negative association between genetically predicted hemoglobin concentration and stroke, including ischemic stroke and cardiogenic stroke. Significant causal relationships were identified using the Inverse Variance Weighted (IVW) method. The results from different MR methods are as follows: Firstly, data from FinnGen demonstrated a significant negative association between hemoglobin concentrations and stroke, with an odds ratio (OR) of 0.82 (95% confidence interval [CI]: 0.73 to 0.92) and a *p*-value of 0.0006. Secondly, findings from MEGASTROKE also reveal a negative correlation between hemoglobin concentration and stroke (OR: 0.91, 95% CI: 0.83–1.00, *p* = 0.040). This association is observed for both ischemic stroke (OR: 0.87, 95% CI: 0.79–0.96, *p* = 0.004) and cardiogenic stroke (OR: 0.82, 95% CI: 0.69–0.99, *p* = 0.039) (detail in Supplementary Table S12). Notably, even after applying Bonferroni correction, hemoglobin concentration remains significantly negatively correlated with ischemic stroke. Thus, it’s revealed that hemoglobin concentration can reduce the incidence of stroke, suggesting that hemoglobin concentration is a protective factor against stroke. The scatter plot indicates that the hemoglobin concentration is negatively correlated with any stroke, ischemic stroke and cardiogenic stroke in the MEGASTROKE database (shown in Figure 3). The funnel plot indicates no heterogeneity between hemoglobin concentration and different stroke subgroups. The leave-one-out plot also indicates the stability of this study model. When hemoglobin concentration is analyzed against stroke in the FinnGen R9 database, there is heterogeneity (*Q* = 181.427, *p* < 0.05),suggesting that our MR-Egger method did not outperform the inverse variance weighting method (16), as is shown in Figure 4.

### 3.2 Exploration of the causal relationship between stroke and hemoglobin concentration

To establish a causal relationship between hemoglobin concentration and stroke, a reverse Mendelian randomization (MR) study is conducted. The reverse MR results indicate no significant association between hemoglobin concentration and the various types of stroke (available in the Supplementary Tables S13–S18). Based on the IVW results, it can be concluded that there is no causal relationship between hemoglobin concentration and different subtypes of stroke. The specific findings are as follows: Any stroke: OR 1.13, 95% CI: 0.90–1.41, *p* = 0.288;Ischemic stroke: OR 1.01, 95% CI: 0.80–1.27, *p* = 0.922;Large artery stroke: OR 0.99, 95% CI: 0.98–1.00, *p* = 0.421; Cardioembolic stroke: OR 1.11, 95% CI: 0.91–1.35, *p* = 0.312. For the Small vessel stroke subtype, an MR analysis was conducted without SNP after applying a strong correlation threshold of *p* < 5e-08. Subsequently, an MR analysis is performed between Small vessel stroke and hemoglobin concentration, setting a *p*-value threshold of *p* < 5e-06. The results of this analysis also show that there is no causal relationship between the two variables (OR: 1.00, 95% CI: 0.99–1.01, *p* = 0.671). These results indicate that there is no significant evidence to suggest a causal relationship between hemoglobin concentration and these specific stroke subtypes. Furthermore, employing bidirectional MR analysis, it is observed that hemoglobin concentration exhibits a negative correlation with stroke, particularly ischemic stroke.

## 4 Discussion

The present MR study of hemoglobin concentration and stroke makes use of the summary statistics of hemoglobin concentration from the UK Biobank consortium, and from the FinnGen R9 and MEGASTROKE consortium for stroke. A two-sample MR analysis is carried out to investigate the potential causal association between these two factors. Novel findings demonstrate a negative causal relationship between hemoglobin concentration and stroke, particularly ischemic stroke. Additionally, the results suggest that hemoglobin concentration acts as a protective factor against stroke, providing the first indication of its potential role in stroke prevention.

A large number of observational studies have examined the relationship between hemoglobin concentration and stroke, but there is no uniform interpretation of their relationship (21). Previous studies have suggested that hospitalized patients with high hemoglobin concentration are more likely to have a stroke than those with low hemoglobin concentration (22). Lee et al. found that an elevation in Hb concentration from the normal range to high levels was associated with an increased risk of stroke (hazard ratio [95% confidence interval]: 1.10 [1.02–1.35]) (23). The increased risk is attributed to high hemoglobin levels causing elevated blood viscosity and a higher propensity for thrombosis. Excess hemoglobin can also damage the endothelium of blood vessels, leading to artery walls thickening and an increased risk of arterial narrowing, all contributing factors to the stroke risk. On the other hand, low hemoglobin concentration can cause anemia, insufficient oxygen supply to the brain and some cardiovascular and cerebrovascular problems. Observational studies have shown an association between low hemoglobin concentrations and a higher likelihood of stroke occurrence (8). Therefore, it is very necessary to elucidate the causal relationship between hemoglobin concentration and stroke. This two-sample Mendelian randomization study shows that hemoglobin concentration is negatively correlated with stroke, challenging the previous view that high hemoglobin concentration is more likely to cause stroke.

Anemia, characterized by low hemoglobin levels, a prevalent condition and recognized as the fifth major cardiovascular risk factor. It affects individuals across all age groups, including children, adults, and the elderly. The relationship between anemia and stroke has been extensively discussed in clinical practice and research, with studies exploring this relationship even in pediatric populations. For example, studies have indicated that low hemoglobin concentration is a major risk factor for increased stroke risk in children with sickle cell anemia (24). Maguire et al. have demonstrated that iron deficiency anemia was a significant risk factor for stroke in otherwise healthy young children (25). The youth population can experience both hypoproliferative anemia and hyperproliferative anemia, which are linked to cerebrovascular diseases. These diseases range from transient ischemic attacks to ischemic stroke and hemorrhagic stroke (26). In adults, low hemoglobin levels have been associated with an increased risk of stroke in both men and women, irrespective of their gender. Panwar et al. demonstrated that the likelihood of stroke in women increased by a factor of 0.59 for every unit decrease in hemoglobin levels (4). The study also indicates that a gradual decline in hemoglobin levels over time may elevate stroke risk, with a Hazard Ratio (HR) of 4.12 (95% Confidence Interval: 1.50, 11.28) in men (27). In the study investigating the connection between chronic kidney disease and stroke, it is found that individuals with anemia had a significantly higher risk of stroke compared to those without anemia (HR 5.43; 95% CI 2.04 to 14.41) (28). Research conducted on the elderly population has demonstrated a negative correlation between hemoglobin levels and stroke. Additionally, it has been found that low hemoglobin status is an independent predictor of both short-term and long-term mortality (29). Another study also shows that decreased in-hospital hemoglobin is independently associated with increased stroke events in older patients (30). The aforementioned study on low hemoglobin concentration and stroke risk further supports the claim that our two-sample Mendelian randomization indicates a negative correlation between hemoglobin concentration and stroke. Raphae S Barlas et al. conducted a meta-analysis on the relationship between anemia and stroke, the study found strong evidence that anemia patients had a higher risk of stroke, with a negative correlation between hemoglobin concentration and stroke incidence (31).

The relationship between hemoglobin concentration and stroke has been observed to exhibit a J or U-shaped curve. This means that the risk of stroke tends to increase at both low and high hemoglobin levels (32–34). However, the present Mendelian randomization study demonstrates that hemoglobin concentration is a protective factor against stroke. Similarly, increasing hemoglobin concentration to the normal range in cases of anemia may also reduce the occurrence of stroke. Earlier studies have also indicated that reducing hemoglobin concentrations from the high range to the normal range reduces the risk of stroke (hazard ratio [95% confidence interval]: 0.80 [0.60–0.97]), and improving anemia to the normal range also reduces the risk of all-cause stroke (hazard ratio [95% confidence interval]: 0.81 [0.69–0.94]) (23).

This study provides a significant advantage as it is the first to establish a causal relationship between hemoglobin concentration and stroke using MR Analysis. Furthermore, it is the first time that hemoglobin concentration is used as a protective factor against stroke. This approach allows doctors to eliminate the influence of confounding factors and establish a reverse causal reasoning. To ensure the reliability of our findings, genetic variation data is collected from the largest available UK Biobank meta-analysis on hemoglobin concentration and from the FinnGen R9 and MEGASTROKE consortium for stroke. The selection of instrumental variables strengthens the MR Analysis. Measures are also taken to detect and exclude horizontal pleiotropy through MR-Egger regression intercept item tests. Additionally, a two-sample MR Design is employed, using non-overlapping exposures and summarizing results at a level data to minimize bias and increase the validity of the conclusions.

There are some limitations of the MR analysis that need to be considered. This study considers the heterogeneity of the included population, taking into account factors such as education level, cultural background, and living habits that may impact the experimental results. Further investigation is needed to explore the causal relationship between education level, living habits, and stroke. Additionally, since the study only includes European individuals, it is important to examine the potential causal relationship between racial differences and stroke, which should be a focus of future research.

## 5 Conclusion

This study conducted a two-sample MR Study and successfully identified a causal relationship between hemoglobin concentration and stroke. Then, with stroke as the exposure factor and Hb as the outcome, we reevaluated the causal relationship between them. The findings indicate a potential protective effect of higher hemoglobin levels against stroke. However, further large sample multi-center randomized clinical studies are needed to validate these results. The study also highlights the importance of considering the impact of hemoglobin concentration on stroke, particularly in the management of anemia patients. This highlights the need for increased attention to hemoglobin levels in the context of stroke prevention and treatment.

## Data availability statement

The datasets presented in this study can be found in online repositories. The names of the repository/repositories and accession number(s) can be found in the article/Supplementary material.

## Ethics statement

The studies involving humans were approved by the Ethics Committee of Longyan First Hospital. The studies were conducted in accordance with the local legislation and institutional requirements. The participants provided their written informed consent to participate in this study. Written informed consent was obtained from the individual(s) for the publication of any potentially identifiable images or data included in this article.

## Author contributions

WW: Funding acquisition, Project administration, Writing – original draft. DF: Data curation, Writing – original draft. BQ: Investigation, Writing – original draft. YC: Writing – review & editing. RQ: Writing – review & editing.

## Footnotes

^1^ http://www.phenoscanner.medschl.cam.ac.uk
