# Supplementary material for: Investigation on the relationship between hemoglobin concentration and stroke risk: a bidirectional Mendelian randomization study
Source: Front Neurol. 2024 Apr 25;15:1327873. doi: 10.3389/fneur.2024.1327873 (PMC11079235; doi:10.3389/fneur.2024.1327873)
Supplement: Supplementary file 8 [file Table_2.DOCX]

| Outcome | Sample size (cases/controls) | Ancestry | Significance level | Data Sources |
| --- | --- | --- | --- | --- |
| Stroke  Stroke  Ischemic stroke  Cardioembolic stroke  Large artery stroke  Small vessel stroke | 39818/271817  40585/406111  34217/406111  7193/406111  4373/406111  5386/192662 | European  European  European  European  European  European | 5e−8  5e−8  5e−8  5e−8  5e−8  5e−8 | <https://www.finngen.fi/fi>r9.finngen.fi  MEGASTROKE Consortium  (ebi-a-GCST005838)  MEGASTROKE Consortium  (ebi-a-GCST005843)  MEGASTROKE Consortium  (ebi-a-GCST005842)  MEGASTROKE Consortium  (ebi-a-GCST005840)  MEGASTROKE Consortium  (ebi-a-GCST005841) |
